# Leak Detection and Localization in Multi-Grid Space Using Improved Gaussian Plume Model

**DOI:** 10.3390/s23136209

**Published:** 2023-07-07

**Authors:** Daquan Li, Gaigai Liu, Zhaoyong Mao

**Affiliations:** 1 Key Laboratory of Unmanned Underwater Vehicle, Ministry of Industry and Information Technology, School of Marine Science and Technology, Unmanned System Research Institute, Northwestern Polytechnical University, Xi’an 710072, China; lidaquan111@163.com; 2 Key Laboratory of Instrumentation Science and Dynamic Measurement, Ministry of Education, North University of China, Taiyuan 030051, China; b200610@st.nuc.edu.cn

**Keywords:** leak detection and localization technology, multi-grid space, gas diffusion model, gaussian plume model, concentration gradient

## Abstract

Leak detection and localization of liquid or gas is of great significance to avoid potential danger and reduce the waste of resources. Leak detection and localization methods are varied and uniquely suited to specific application scenarios. The existing methods are primarily applied to conventional pressurized pipelines and open areas, and there are few methods suitable for multi-grid spaces. In this paper, a gas diffusion model applied to multi-grid space is constructed, and a method for leak detection and localization using the concentration gradient of characteristic gas is proposed according to the prediction behavior. The Gaussian plume model is selected due to its advantages of simplicity and the interpretation of gas diffusion behavior is closer to reality; the expression of the improved model is also obtained. To verify the correctness of the model and the applicability of the localization method, taking the coolant leakage in the circuit system as an example, three experiments with different source strengths were repeated. The fitting correlation coefficients between the gas concentration data of the three experiments and the model are 0.995, 0.997 and 0.997, respectively. The experimental results show that the model has a strong correlation with the real plume behavior, and it is reasonable to use the gas concentration gradient for the localization of the leak source. This study provides a reference for future research on the leak detection and localization of gas- or liquid-containing volatile substances in a complex multi-grid space.

## 1. Introduction

The transmission and storage of liquid or gas are widely used in the chemical, electric power, food and medical industries, as well as other fields [1,2]. The leakage will not only cause economic loss and waste of resources [3], but the toxic gas released will also cause environmental pollution, explosion, fire, etc. [4]. Therefore, leak detection and localization technology have attracted extensive attention. In the past few decades, various leak detection and localization methods have been proposed, and are uniquely suitable for applications in specific scenarios [5,6].

Ge et al. [7] proposed a negative pressure wave method for leak detection and localization of pressurized pipelines, which was widely used in the energy distribution industry [8]. Guo et al. [9] used an exclusive frequency domain analysis method to achieve leak detection in water distribution systems. Ranginkaman et al. [10] studied the application of the frequency response method in the looped pipe networks, showing that it has the advantages of fast calculation and reliability in the transient flow analysis of the pipeline network. Li et al. [11] presented a method combining the acoustic emission technique and artificial neural network-based pattern recognition to achieve the leak detection and localization of water distribution pipe subject to failure of the socket joint, and the results showed that the estimation accuracy reached 97.2% and 96.9%. Obviously, the above methods are all proposed for the leak detection and localization of conventional pressurized pipelines. In addition, methods such as visual inspection, electromagnetic detection, ultrasound detection [12], infrared thermal imaging [13], stress wave detection [14] and real-time flow modeling [1,15] are also used for the leak detection and localization of pressurized pipelines. These methods detect leaks based on the changes in physical parameters, such as pressure, flow and acoustic characteristics in the pipeline, and have certain limitations when used in areas surrounded by electromagnetic sources, vibration, and other causes of interference. Moreover, the localization methods are challenging in terms of meeting the application requirements in areas with complex spatial structures.

In addition, there are some leak detection and localization technologies applied to open areas, such as the leak detection technology based on polymer cable and the leak detection technology based on distributed optical fiber [16], which can be arbitrarily arranged in areas with complex spatial structures. However, the polymer cable-based leak detection technology suffers from the issue of condensation and insufficient positioning accuracy. Its detection distance is hundreds of meters and its positioning accuracy is 1% ± 0.5 m of the length of the sensing element, thus higher positioning accuracy is difficult to achieve. Compared with leak detection based on polymer cable, distributed optical fiber detection has a certain improvement in positioning accuracy, but the real-time performance is not strong, and the response time is as long as several minutes [17,18,19]. Moreover, within the distributed optical fiber detection exists the problem of temperature and noise cross-sensitivity. Therefore, various types of methods have their advantages and limitations when applied to different scenarios.

Coolant is usually used in large-scale circuit systems, such as large data centers and phased array radars, to solve the heat dissipation problem [20]. In these application scenarios, the leakage space is divided into many narrow grid units, and the electronic devices are densely distributed in the grid space; a tiny leakage may cause serious consequences and threaten the circuit system. Moreover, there are a large number of tested positions, and the electromagnetic interference is complex. All of these increase the difficulty of applying traditional pressure pipeline and open area leak detection and localization methods in this complex drawer multi-grid space.

We know that the coolant usually contains volatile characteristic substances. When a leak occurs, the leakage state is divided into two stages: early small leakage with low gas concentration and late large leakage with high gas concentration. Therefore, the corresponding leak detection methods include liquid-based detection and gas-based detection. The liquid-based detection belongs to contact-type detection, which requires the leaked liquid to flow to the surface of the sensor before an alarm is issued. However, at this time, the liquid may have flooded part of the circuit structure and caused losses. Therefore, the safety of circuit systems cannot be fundamentally guaranteed. In fact, the aforementioned leak detection and localization methods for pressurized pipelines and open areas are all contact-type detection methods. Compared with liquid-based detection, non-contact gas-based detection can monitor the small leakage in early failure in a timely manner, effectively avoiding the serious consequences caused by massive leakage. Sanchez-Sosa et al. [21] studied the gas diffusion model in an empty room in detail and applied the model to realize the leak source localization. However, this method only studies the detection and localization of a leak in a single confined space, and it is not applicable to complex multi-grid confined spaces. Wang et al. [22] studied the diffusion process of gas leaking from buried natural gas pipelines into the adjacent confined spaces through the soil, obtained the gas concentration prediction model and established a hazardous boundary calculation model of the adjacent confined spaces, but they did not locate the leak source. In fact, there have been few studies on the methods of leak detection and localization in complex drawer multi-grid confined spaces [20,23,24].

In order to fill in the research gap, this paper proposes a leak detection and localization method applied to complex drawer multi-grid confined spaces. Taking the coolant leak in large-scale circuit systems as an example, we make corresponding assumptions and establish a gas diffusion model applied to gridded confined spaces. Then, according to the predicted plume behavior, a concentration gradient method is proposed to achieve the localization of the leak source. In the process of leak detection and localization, only the gas concentration data collected by the array-distributed metal oxide gas sensors are used, and the detection of the liquid leak is converted into gas concentration-based detection, which realizes the non-contact detection and early detection of leakage behavior. The experimental results prove that the proposed model is consistent with the gas diffusion behavior in the actual multi-grid space, and show the feasibility of applying the concentration gradient to the localization of the leak source in the gridded confined space. The proposed method can be used not only for the leak detection and localization of liquids containing volatile substances, such as coolants, but also for the leak detection and localization of pure gases.

## 2. Principle

### 2.1. Traditional Gaussian Plume Model

Atmospheric dispersion models have been developed for decades to understand the behavior of pollutants thrown into the atmosphere [21]. The existing gas diffusion models include the Gaussian model [25], similarity-profile models [26] and computational fluid dynamics (CFD) models [27]. Due to its simplicity and explanation of the diffusion behaviors being close to reality, the Gaussian model has been widely used. The traditional Gaussian model is suitable for the study of the diffusion of the gas, the density of which is close to the air or the density is close to the air after a short period of air dilution, including the Gaussian puff model and the Gaussian plume model [28]. Generally, the Gaussian puff model is used for the simulation of large-scale gas leakage and diffusion over a short period of time, whereas the Gaussian plume model is used for the simulation of continuous gas leakage and diffusion over a long period of time [29]. At present, most of the coolants used in large-scale circuit systems are ethylene glycol-type coolants, the main component of which is ethylene glycol. Ethylene glycol is colorless, odorless, has low-volatility and is toxic, and is one of the volatile organic compounds (VOCs). Once the coolant leaks, it will not rapidly spread in a short period of time. Therefore, the form of the leak is a continuous leak, which can be simulated by the Gaussian plume model.

**Figure 1:**
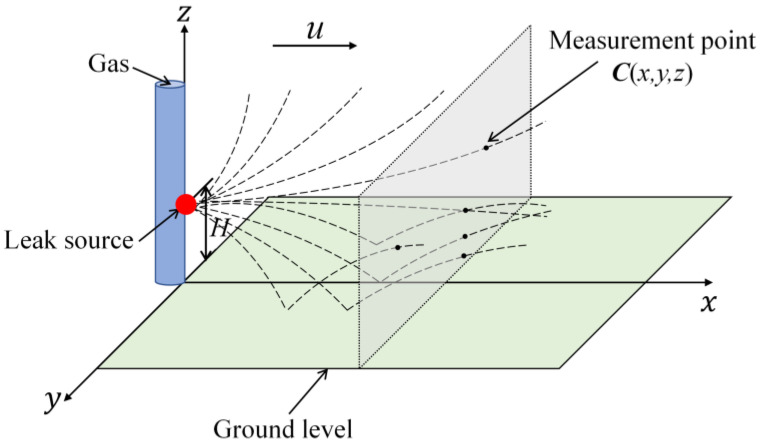
A sketch map of traditional Gaussian plume model.

A sketch map of the plume behavior predicted by the traditional Gaussian plume model is shown in Figure 1. A Cartesian coordinate system is established with the projection of the gas source on the ground surface as the origin, the positive direction of the *x*-axis is consistent with the wind direction, and the positive direction of the *z*-axis is perpendicular to the ground level and upwards. Turner et al. [30,31,32,33] gave the expression of the Gaussian plume model as follows:

(1) $$C(x,y,z,H)=\frac{Q}{2\pi u\sigma_{y}\sigma_{z}}exp\left(-\frac{y^{2}}{2\sigma_{y}^{2}}\right)\left\{exp\left[-\frac{{\left(z-H\right)}^{2}}{2\sigma_{z}^{2}}\right]+\alpha exp\left[-\frac{{\left(z+H\right)}^{2}}{2\sigma_{z}^{2}}\right]\right\}$$

where *C* represents the concentration (mg/m^3^) of the gas at measurement point (*x*, *y*, *z*) (m), *x* is the distance from the leak source to the measurement point in the down-wind direction, *y* is the distance from the leak source to the measurement point in the cross-wind direction, *z* is the distance from the ground level to the measurement point in the vertical wind direction; *H* is the height (m) of the leak source above the ground level; *Q* is the strength (mg/s) of the leak source, which is a constant; *u* is the wind speed (m/s) in the environment, which is also a constant; *σ_y_*, *σ_z_* are the coefficients of diffusion, affected by the level of atmospheric stability, and finally determined by *x* and *u* [21,34]; *α* is the proportion of the material reflected back into the plume when it reaches the ground surface [31] (for perfectly absorbing ground surface *α* = −1, whereas for perfectly reflecting ground surface *α* = 1 [33,35]).

Although the behavior of the plume at one moment is random and unpredictable, after a period of sampling, the random behavior can be exchanged for a uniform behavior around the mean [33], so the prediction is valid. The traditional Gaussian plume model is used in open areas where gas can diffuse over several kilometers [32]. When applied to confined spaces, it has limitations due to the interaction of the plume with surrounding walls, soil and ceilings [21]. In addition, the traditional Gaussian plume model is effective for the application scenarios to understand the concentration distribution of gases emitted from point sources into the atmosphere subjected to a unidirectional wind, and it is not suitable for no wind conditions (namely, *u* = 0).

### 2.2. Improved Gaussian Plume Model

In order to realize leak detection and localization in the multi-grid confined space, we take the coolant leakage in a large-scale circuit system as an example to make corresponding assumptions about the environmental conditions, and construct a gas dispersion model suitable for the multi-grid space by improving the traditional Gaussian plume model. We know that the internal environment of the multi-grid space is complex, and many units are densely arranged. Each unit can be considered an independent diffusion space. The structure between adjacent units is not sealed, there are narrow gaps or holes that allow gas to pass through. Once the coolant in the large-scale circuit system leaks, except for the unit where the leak source is located, the gas in the remaining units comes from the pores on the four walls that make up the unit. Although the gas plume behavior in a single unit is relatively complex due to the collision, flow around and swirl caused by the interaction between the gas and the six confinement surfaces, the gas concentration distribution in the overall grid space can be considered as a Gaussian distribution with uniformity and symmetry. Therefore, a model for the gas concentration distribution in the overall grid space can be established. In addition, due to the obstruction of the wall, the diffusion rate of gas in the multi-grid space is much lower than that in the open area. Therefore, there is an obvious concentration difference between the unit where the gas source is located and the units around it. According to this principle, the leaking unit can be easily determined.

To establish the gas diffusion model applied to a multi-grid space, we make the following assumptions on the basis of the traditional Gaussian plume model according to the real environment of coolant leakage in the large-scale circuit system:

(1) The leak source is located at the ground level, thus *H* = 0.
(2) The diffusion space of *z* < 0 is exactly the same as the diffusion space of *z* > 0; that is, the diffusion area is symmetrical based on the leakage source, and the ground surface neither absorbs nor reflects; i.e., *α* = 0.
(3) The environmental conditions are stable and the diffusion coefficient is isotropic; that is *σ_y_* = *σ_z_* = *σ.*

Then, Equation (1) becomes:

(2) $$C\left(x,y,z\right)=\frac{Q}{2\pi u\sigma^{2}}exp\left(-\frac{y^{2}+z^{2}}{2\sigma^{2}}\right)$$

According to the real environment of coolant leakage in the large-scale circuit system, the leak space is gridded and divided into many narrow drawer units. We select the plane with constant *x* (*x* > 0) as the measurement plane (assuming *x* = *x*_0_), and the measurement points are evenly distributed in an array on the gridded measurement plane. Then, the gas concentration at any measurement point in the planar measurement array can be described as:

(3) $$C\left(x_{0},n_{y},n_{z}\right)=\frac{A}{2\pi {\left(\frac{\sigma }{d}\right)}^{2}}exp\left(-\frac{n_{y}{}^{2}+n_{z}{}^{2}}{2{\left(\frac{\sigma }{d}\right)}^{2}}\right),A=\frac{Q}{vSd^{-1}}$$

where *n_y_* = …, −2, −1, 0, 1, 2, …, *n_z_* = …, −2, −1, 0, 1, 2, … represent the discrete coordinates of the unit where the measurement point is located in the cross-wind direction and vertical wind direction, respectively, with the unit where the leak source is located as the origin; *v* is the gas molecular diffusion coefficient (m^2^/s); *S* is the area (m^2^) of the pores on the wall of the unit for the gas to pass through; *d* is the distance (m) between adjacent measurement points, that is, the side length of the unit in the grid space. Since the grid space is less affected by the atmospheric conditions, the diffusion coefficient *σ* is determined by *x*_0_ and *v*.

**Figure 2:**
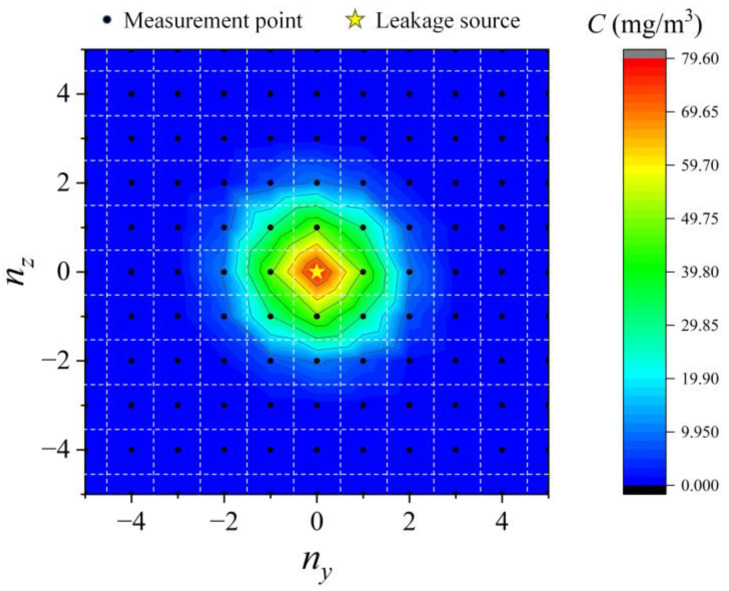
The isoconcentration line of the concentration surface on the gridded measurement plane.

The isoconcentration lines of the concentration surface defined by Equation (3) on the gridded measurement plane are shown in Figure 2, with the values *x*_0_ = 0.1 m, *Q* = 0.001 mg/s, *v* = 1 × 10^−4^ m^2^/s, *d* = 0.5 m, *S* = 0.01 m^2^ and *σ* = 0.5. As expected, the position where the gas concentration and concentration gradient are greatest corresponds to the location of the gas source.

Then, define *R*^2^ = *n_y_*^2^ + *n_z_*^2^, and substitute it into Equation (3), we can obtain:

(4) $$C(x_{0},R)=\frac{A}{2\pi \sigma_{1}{}^{2}}exp\left(-\frac{R^{2}}{2\sigma_{1}{}^{2}}\right),\sigma_{1}=\frac{\sigma }{d}$$

which indicates the relationship between the gas concentration *C* of the measurement point in the gridded measurement plane and the distance *R* between the measurement point unit and the leak source unit. According to Equation (4), theoretically, the gas concentrations of the measurement points with the same *R* are equal at the same moment.

**Figure 3:**
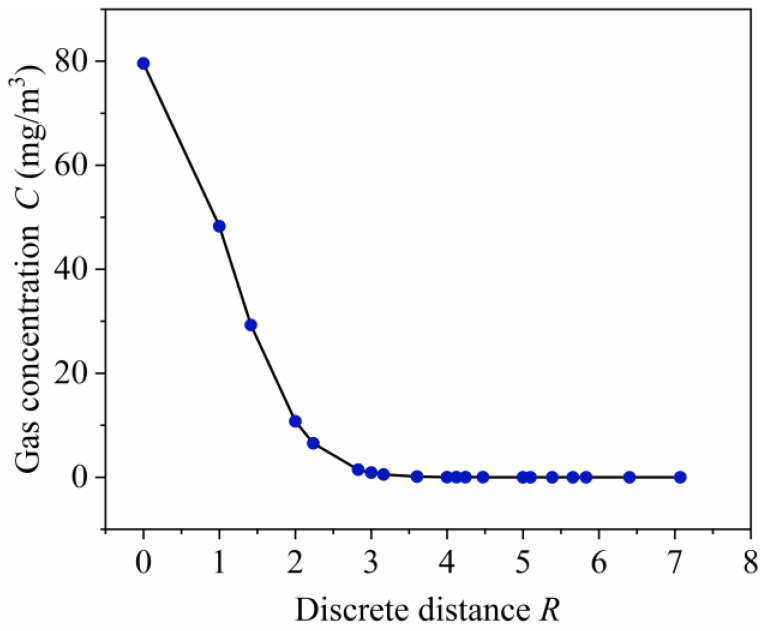
Function curve of Equation (4).

Figure 3 shows the graph of Equation (4), from which we can find that the position where the concentration and concentration gradient is maximum is exactly where the leak source is located. Thus, the localization problem of the leakage source can theoretically be simplified from 2D to 1D.

In practice, the diffusion behavior of gas in an open area is easily affected by factors such as airflow in the environment and the mechanical structure of the leak source. Therefore, the gas concentration at a measurement point in an open area is not only affected by the distance. However, in the multi-grid space, due to the obstruction of the unit wall and the stability of the airflow inside the grid space, there is an obvious relationship between the gas concentration of the measurement point and the distance between the measurement point unit and the leak source unit. Equation (4) gives an expression for this relationship. From Equation (4), we can know: the farther the distance, the smaller the concentration of the measurement point; the farther the distance, the longer it takes for the sensor installed on the measurement point to reach the peak value of the concentration.

The improved model has practical significance for leak detection and localization applications in complex multi-grid confined spaces. On the one hand, if the location of the leak source is known, the distribution of gas concentration in the grid space can be predicted according to the model. On the other hand, by periodically scanning the concentration of each measurement point in the planar measurement array and drawing the concentration distribution map, the position with the largest concentration and concentration gradient can be determined as the location of the leak source; thus, the leak source localization can be achieved. In addition, the improved gas diffusion model also addresses the shortcoming of the traditional Gaussian plume model that takes wind velocity as a necessary condition.

## 3. Experimental Settings

**Figure 4:**
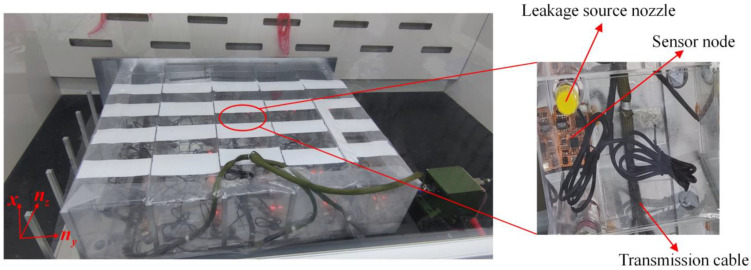
Photo of the leak detection and localization experiment in a simulated multi-grid space.

In order to verify the correctness of the model and the feasibility of the concentration gradient localization method, a 5 × 5 grid confined space was established according to the real environment of the coolant leakage in a large-scale circuit system. As shown in Figure 4, the length, width and height of the grid space were 75 cm, 75 cm and 15 cm, respectively, and the volume of each unit was 15 cm × 15 cm × 15 cm. The area of the pores on the unit wall for gas to pass through was fixed at 0.4 cm × 15 cm × 4. Just like the actual large-scale circuit system, the internal space of each unit was narrow, so it was necessary to use small-volume devices for measurements. The CCS811 metal-oxide gas sensors produced by Cambridge CMOS Sensors Company were selected for the measurement of the gas concentration. The sensor has the characteristics of full digitization, high sensitivity and low power consumption. Furthermore, it adopts MEMS packaging technology, the package size is 2.7 mm × 4.0 mm, which meets the installation requirements of narrow spaces. In addition, since the temperature, humidity and air pressure inside the grid space also affects the diffusion behavior of gas, the sensor nodes were integrated with temperature, humidity and air pressure sensors for real-time monitoring and compensation. In order to adapt to the special-shaped structure in the large-scale circuit system, the acquisition board adopted a flexible board and was installed by surface mounting. The transmission of data adopted a new generation of SharkNet data transmission bus with reliability and high real-time performance.

Before the experiment, the MEMS metal oxide sensor was installed in the position where the leak was most likely to occur in each unit, and 25 sensors were evenly distributed in the grid space to form a distributed measurement system. We selected a unit where a leak event was about to occur, and installed a special nozzle in it. During the experiment, No.65 coolant was injected into the unit through the nozzle to simulate the leak of coolant in the circuit system. After the leakage occurred, the characteristic gas of ethylene glycol in the coolant naturally volatilized, firstly detected by the high-sensitivity gas sensor in the leaking unit, and then diffused to the surrounding units through the pores on the wall. The 25 sensor nodes transmitted the collected data to the processor in real time, and the processor packed the data and uploaded it to the host computer through the SharkNet bus to realize the visual display of the concentration data and the localization of the leak source.

## 4. Results and Discussion

### 4.1. Variation of Gas Concentration at Different Distances

**Figure 5:**
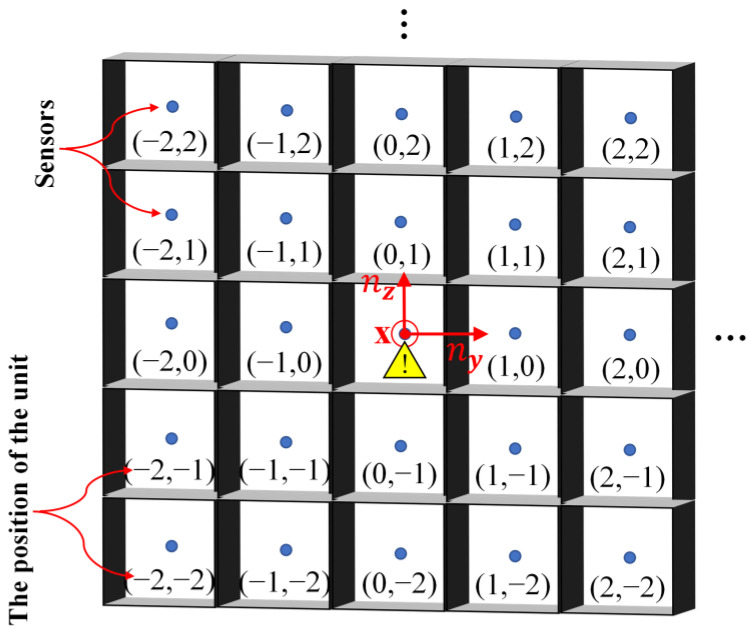
A sketch map of the structure of the multi-grid space.

**Figure 6:**
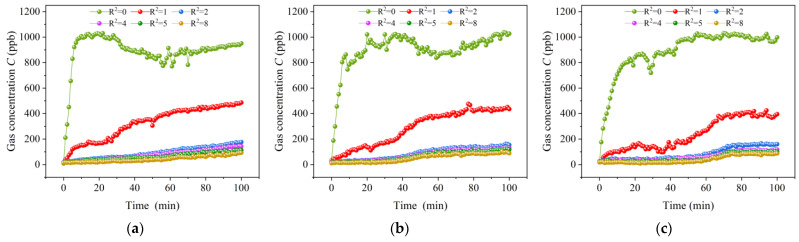
The variation in gas concentration with time at different distances R when the strength of the leakage source is 3 × 10^−5^ mg/s, 2 × 10^−5^ mg/s and 1 × 10^−5^ mg/s, respectively: (**a**) Q = 3 × 10^−5^ mg/s; (**b**) Q = 2 × 10^−5^ mg/s; (**c**) Q = 1 × 10^−5^ mg/s.

As shown in Figure 5, for ease of description, we abstracted the grid space and numbered each unit in the form of (*n_y_*, *n_z_*) coordinates. The unit located at the center of the grid space was selected as the unit where the coolant leak was about to occur, and was set as the origin with coordinates (0,0). To ensure the validity of the experiment, three repeated experiments were carried out with only the strength of the leak source changed. The variation of the gas concentration in each unit was recorded from the moment of leakage. The total recording time was 100 min with an interval of 1 min. Then, we calculated the distance between the measurement point unit and the leak source unit according to the equation $R=\sqrt{n_{y}{}^{2}+n_{z}{}^{2}}$, and took the average value of the gas concentration of the units with the same *R* as the gas concentration value as at that *R*. Figure 6a–c shows the variation in gas concentration at different distance *R* when the strength of the leakage source was 3 × 10^−5^ mg/s, 2 × 10^−5^ mg/s and 1 × 10^−5^ mg/s, respectively.

From Figure 6a–c, it can be seen that, regardless of the strength of the leak source, once the leak occurs, the metal oxide gas sensor in the unit where the leak source is located can immediately respond to the change in the concentration of ethylene glycol gas and rapidly increase. With the increase in the distance *R*, the response time becomes longer and the gas concentration increases more slowly. When the strength of the leak source is 3 × 10^−5^ mg/s, the concentration of the unit at *R*^2^ = 0 increases by 202 parts per billion per mol (ppb) within one minute after the leak occurs, while the concentration of the units at *R*^2^ = 1,2,4,5,8, increases by 22, 9, 9, 8, 3 ppb, respectively. Therefore, an obvious concentration difference is formed between the leak source unit and its surrounding units. The gas concentration rapidly decreases with the increase in the distance *R*, which is consistent with the Gaussian model.

Comparing the three experiments, we found that before reaching the concentration peak, at the same distance *R*, the greater the strength of the leakage source, the higher the gas concentration at the same moment; that is, the gas concentration is proportional to the strength of the leak source, which is consistent with the improved Gaussian plume model. In addition, at the same distance *R*, the greater the strength of the leak source, the faster the concentration increases and the faster the time to reach the peak of the concentration. When the strength of the leak source is 3 × 10^−5^ mg/s, the gas concentration in the unit where the leak source is located reaches the concentration peak at about 19 min. However, when the strength of the leak source is 2 × 10^−5^ mg/s and 1 × 10^−5^ mg/s, the gas concentration of the leak source unit reaches the peak at 36 min and 55 min, respectively. After reaching the peak, the growth rate of the gas concentration in the unit slows down and maintains a slight fluctuation around the peak, and the gas begins to diffuse to the surrounding units in large quantities through the pores. At the same time, the gas concentration in the surrounding units gradually increases, the growth rate increases and the concentration difference between the unit where the leakage source is located and its surrounding units begins to decrease, which indicates that there is an optimal time domain for gas source localization using the concentration gradient.

### 4.2. Model Validation

**Figure 7:**
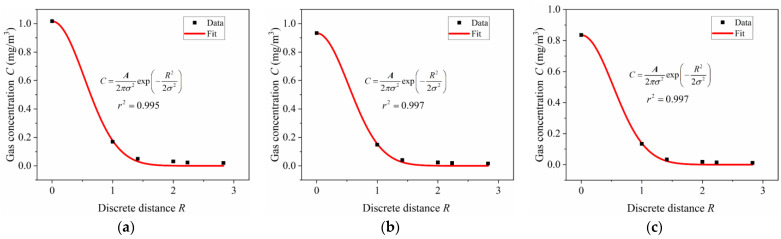
Fitting results of three experiments using Equation (4): (**a**) Q = 3 × 10^−5^ mg/s; (**b**) Q = 2 × 10^−5^ mg/s; (**c**) Q = 1 × 10^−5^ mg/s.

To verify whether the gas concentration distribution in the multi-grid space after the leak occurs conforms to the improved Gaussian plume model, we selected the gas concentration values at 20 min after the leak and fitted it with the improved Gaussian plume model. Since the gas diffusion behavior at a certain moment is random and irregular, it is one-sided to select the value at a certain moment for analysis. We take the average value of the gas concentration at 17–23 min as the gas concentration at 20 min and plot the relationship curve between *R* and *C*, using the Levenberg-Marquardt algorithm to perform the nonlinear curve fitting with Equation (4). The fitting results of the three experiments are shown in Figure 7a–c, and the fitted parameters are shown in Table 1.

The fitting correlation coefficients *r*^2^ of the three experiments are 0.995, 0.997 and 0.997, respectively. The fitting results show that the diffusion behavior of gas in the multi-grid space is consistent with the improved Gaussian plume model; that is, the experimental results are consistent with the theoretical model, which confirms the applicability of the model in the multi-grid confined space.

**Figure 8:**
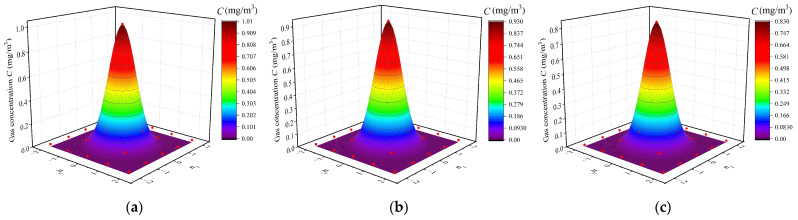
Concentration distributions of the three experiments and fitting results using Equation (3): (**a**) Q = 3 × 10^−5^ mg/s; (**b**) Q = 2 × 10^−5^ mg/s; (**c**) Q = 1 × 10^−5^ mg/s.

Then, we used Equation (3) to perform nonlinear surface fitting on the concentration distribution on the gridded measurement plane. The results are shown in Figure 8a–c. It can be seen that the location where the concentration and the concentration gradient are maximum match the location of the leak source (*R* = 0). Therefore, it is feasible to obtain a concentration distribution map by periodically scanning the concentration of each measurement point and locating the leak source unit according to the maximum concentration and concentration gradient. Since the grid space discretizes the concentration distribution, finite differences can be used instead of gradients.

### 4.3. Positioning Analysis

The correctness of the improved Gaussian plume model is verified above, and the advantages of simplicity, convenience and non-contact measurement of the proposed leak detection method applied to gridded confined space are obvious. Then, we conducted experiments to verify the positioning accuracy and real-time performance of the proposed concentration gradient localization method.

**Figure 9:**
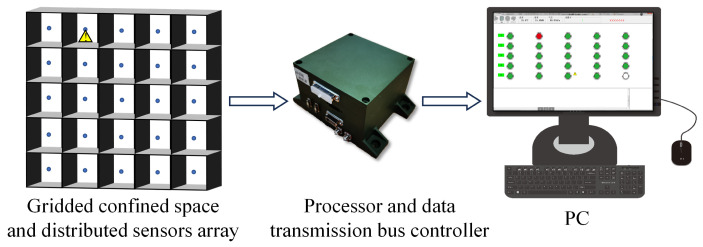
The composition of the leak detection and localization system.

Figure 9 shows the composition of the leak detection and localization system. The specific implementation process of the leak detection and localization method is described as follows:

The processor periodically scans and records the concentration of each measurement point to obtain the concentration distribution map. The unit with the highest gas concentration is set as the origin. Then, take the average value of the gas concentration of the units with the same *R* as the gas concentration value as *R*. Calculate the concentration gradient at each *R* (use finite differences instead of gradients). If the location with the maximum concentration gradient is consistent with the location with the maximum concentration (*R* = 0), then the measurement point is preliminarily considered to be the location of the leak source. If the concentration and concentration gradient of the measurement point maintain the maximum in three consecutive distribution maps, and the gradient value of each time is greater than 100 ppb, then it will be determined as the leak point. 

Under the same experimental conditions, ten repeated experiments were carried out with only the location of the leak source changed. The experimental results showed that the localization success rate of the proposed method was up to 99.99%. In addition, the method could pinpoint the leak source location to a single grid unit regardless of the size of the unit, which demonstrates that the positioning accuracy can reach the centimeter level. A CCS811 metal oxide sensor is a digital gas sensor that integrates an Analog-to-Digital converter (ADC) and a microcontroller unit (MCU) for data acquisition and calculation. In the experiment, we set the measurement cycle time as 1 s. The average localization time of ten repeated experiments was less than 1 min. In fact, the proposed method will immediately trigger leak detection and localization when there is a small leak with gas concentration changes in the early stage, so the real-time performance is definitely higher than that of liquid-based contact-type detection methods. Therefore, the proposed method has the advantages of a high localization success rate, a fast speed and high localization accuracy when applied to leak detection and localization in gridded confined space.

## 5. Conclusions

Most of the existing leak detection and localization methods are proposed for traditional pressurized pipelines and open areas, which have limitations when applied to multi-grid confined spaces, such as large data centers and phased array radar. Research on the prediction of gas diffusion behavior and the leak detection and localization methods in multi-grid space is still very scarce. Taking the leak detection and localization of coolant in a large-scale circuit system as the background, we made corresponding assumptions regarding the environmental conditions and constructed a gas diffusion model suitable for a multi-grid space. The relationship between the gas concentration of the measurement points and the distance from the measurement point unit to the leak source unit was studied. A method to locate the leak source by using the gas concentration gradient was also proposed. In order to verify the correctness of the model and the feasibility of the concentration gradient localization method, we simulated the actual environment of the coolant leakage in a large-scale circuit system and established a multi-grid space for experiments. By fitting the gas concentration data collected by the evenly distributed metal oxide gas sensor, it was proved that the improved model was consistent with the actual gas diffusion behavior in the multi-grid space; therefore, it is reasonable to use the concentration gradient to locate the leak source.

The main motivation of our research was to perform dynamic comparison and analysis according to the variation of the gas concentration in the grid units to obtain the real position of the leak source. The improved Gaussian plume model and the proposed concentration gradient localization method are not limited to applications in large-scale circuit systems; they are also applicable to complex grid environments with risks of gas or liquid leakage in the chemical industry and military and medical fields, such as medical or chemical cabinets. Therefore, the proposed and experimental verification of the gas diffusion model and localization method has practical value. However, only the prototype verification of leak detection and localization under ideal conditions was performed, and there may still be a certain gap when applied to practical engineering applications. Situations such as inconsistent grid unit sizes and multiple leak sources at the same time have not been fully studied. The verification of leak detection and localization capabilities has only been carried out in a small number of grid units, and there may be more practical applications. Therefore, there is still room for improvement. In the future, we will strive to integrate it within engineering practices to solve more engineering problems.

## Figures and Tables

**Table 1:** The fitted parameters.

Parameter	(a)	(b)	(c)
*A*	1.8247	1.62924	1.45763
*σ*_1_	0.53463	0.52725	0.52712

## Data Availability

Not applicable.
